# Diverse Single-Stranded DNA Viruses Identified in Chicken Buccal Swabs

**DOI:** 10.3390/microorganisms9122602

**Published:** 2021-12-16

**Authors:** Klaudia Chrzastek, Simona Kraberger, Kara Schmidlin, Rafaela S. Fontenele, Arun Kulkarni, Len Chappell, Louise Dufour-Zavala, Darrell R. Kapczynski, Arvind Varsani

**Affiliations:** 1 Exotic and Emerging Avian Viral Disease Research Unit, Southeast Poultry Research Laboratory, U.S. National Poultry Research Center, Agricultural Research Service, USDA, 934 College Station Road, Athens, GA 30605, USA; klaudia.chrzastek@gmail.com; 2 The Biodesign Center of Fundamental and Applied Microbiomics, Center for Evolution and Medicine, School of Life Sciences, Arizona State University, 1001 S. McAllister Ave, Tempe, AZ 85287, USA; simona.kraberger@asu.edu (S.K.); kara.schmidlin@asu.edu (K.S.); rafasfontenele@asu.edu (R.S.F.); 3 Georgia Poultry Laboratory Network, 3235 Abit Massey Way, Gainesville, GA 30507, USA; akulkarni@gapoultrylab.org (A.K.); lchappell@gapoultrylab.org (L.C.); lzavala@gapoultrylab.org (L.D.-Z.); 4 Structural Biology Research Unit, Department of Integrative Biomedical Sciences, University of Cape Town, Rondebosch, Cape Town 7700, South Africa

**Keywords:** single-stranded DNA viruses, chicken, respiratory tract, *Cressdnaviricota*, *Microviridae*

## Abstract

High-throughput sequencing approaches offer the possibility to better understand the complex microbial communities associated with animals. Viral metagenomics has facilitated the discovery and identification of many known and unknown viruses that inhabit mucosal surfaces of the body and has extended our knowledge related to virus diversity. We used metagenomics sequencing of chicken buccal swab samples and identified various small DNA viruses with circular genome organization. Out of 134 putative circular viral-like circular genome sequences, 70 are cressdnaviruses and 26 are microviruses, whilst the remaining 38 most probably represent sub-genomic molecules. The cressdnaviruses found in this study belong to the *Circoviridae*, *Genomoviridae* and *Smacoviridae* families as well as previously described CRESS1 and naryavirus groups. Among these, genomoviruses and smacoviruses were the most prevalent across the samples. Interestingly, we also identified 26 bacteriophages that belong to the *Microviridae* family, whose members are known to infect enterobacteria.

## 1. Introduction

High-throughput sequencing (HTS) has emerged as a promising tool for the detection and discovery of known and novel infectious agents in clinical samples. HTS-based approaches provide an alternative solution to conventional culture-based methods for rapid pathogen identification without prior sequence knowledge. A large proportion of novel viruses have been discovered directly from humans and animal clinical samples using HTS-based approaches [1,2,3], but this has also raised issues of contamination with viral-like sequences from lab reagents and during library preparation [3,4,5,6]. However, with appropriate controls and verification, this approach yields robust data.

Over the last several years, viral metagenomics has facilitated the discovery of hundreds of highly divergent single-stranded DNA (ssDNA) viruses that belong in the phylum *Cressdnaviricota* [7], also commonly referred to as circular replication-associated protein encoding single-stranded (CRESS) DNA viruses [3,8,9,10,11,12]. In addition, a large number of ssDNA bacteriophages in the family *Microviridae* [13,14] have also been identified [15,16,17,18,19,20].

The poultry industry is an economically important sector of animal farming globally, and in the US, it was worth over 29 billion dollars in 2020 (https://www.uspoultry.org/economic_data/) (accessed in 15 February 2021). Diseases caused by viral infections can result in high mortality and, thus, have huge economic consequences for the industry. Therefore, concerted efforts and resources are directed at monitoring poultry health, including virus infections. Poultry are regularly monitored for influenza A virus, which can have an impact on human and animal health globally. Many different viruses representing many viral families have been identified (Table 1) in commercial poultry. [21,22,23,24]. In addition, several viruses from the ssDNA viral families including *Anelloviridae*, *Circoviridae*, *Genomoviridae*, *Parvoviridae* and *Smacoviridae* have been previously identified (Table 1).

To build on the current knowledge of viruses found in poultry, we performed HTS of DNA extracted from buccal swabs of chickens. In these, we were able to identify novel unclassified cressdnaviruses as well as viruses that are part of the *Circoviridae*, *Genomoviridae* and *Smacoviridae* families. Furthermore, we also identified bacteriophages that are part of the *Microviridae* family.

## 2. Materials and Methods

### 2.1. Sample Collection and Processing

Six buccal swab samples of randomly selected chickens (30-week-old mixed breeders) from a farm located in Georgia, USA were individually collected in sterile tubes and stored in brain heart infusion (BHI) broth (Sigma, St. Louis, MO, USA). The BHI broth was then filtered through a 0.2 μm filter and the filtrates were immediately stored at −20 °C. DNA was isolated using the QIAamp DNA Blood Mini Kit (Qiagen, Germantown, MD, USA).

### 2.2. Illumina MiSeq Sequencing

Whole genome amplification (WGA) was performed on the extracted DNA using the Illustra GenomiPhi V2 DNA Amplification kit (GE Healthcare, Chicago, IL, USA) as per the manufacturer’s protocol. The WGA DNA was purified using the Agencourt AMPure XP beads (Beckman Coulter, Pasadena, CA, USA) at a ratio of 0.7× to select DNA fragments >500 bp in size. For quantification of the dsDNA, the Qubit dsDNA HS assay (Invitrogen, Waltham, MA, USA) was used. A quantity of 1 ng of DNA was used to generate multiplexed paired-end sequencing libraries using the Nextera XT DNA Sample Preparation Kit (Illumina, San Diego, CA, USA). The dsDNA was fragmented and tagged with adapters using Nextera XT transposase (Illumina, San Diego, CA, USA). The Nextera XT transposome fragmented PCR amplicons with added adaptor sequences enabled a 12-cycle PCR amplification to append additional unique dual index (i7 and i5) sequences at the end of each fragmented DNA for cluster formation. PCR fragments were purified with Agencourt AMpure XP beads (Beckman Coulter, Pasadena, CA, USA). Fragments were analyzed on a High-Sensitivity DNA Chip using the Bioanalyzer (Agilent Technologies, Santa Clara, CA, USA). The final concentration of the library pool was diluted to 10 pM. A control library (3% PhiX library, Illumina, San Diego, CA, USA) was added and the pool was snap-chilled on ice. The library pool was loaded in the flow cell of the 500 cycle MiSeq Reagent Kit v2 (Illumina, San Diego, CA, USA) and pair-end sequencing (2 × 250 bp) was performed on the Illumina MiSeq instrument (Illumina, San Diego, CA, USA).

### 2.3. Identification of DNA Viruses and Determination of Complete Viral Genomes

The quality of sequencing reads was assessed using FastQC ver. 0.11.5 [25], and the reads were then quality trimmed with a Phred quality score of 30 or more, in addition to low-quality ends trimming and adapter removal using Trim Galore ver.0.5.0 (https://www.bioinformatics.babraham.ac.uk/projects/trim_galore/) (accessed in 15 February 2021).The reads were *de novo* assembled using metaSPAdes 3.12.0 [26] with k = 33, 55, 77. The *de novo* assembled contigs >750 nt in length were analyzed against a viral protein RefSeq database using BLASTx [27]. Circular molecules were identified among the viral-like sequences by checking for terminal redundancy. The open reading frames in the viral genomes were determined with ORFfinder (https://www.ncbi.nlm.nih.gov/orffinder/) (accessed in 15 February 2021) and manually checked and annotated. The viral genomes identified in this study were deposited in the GenBank database (accession numbers: MN379584–MN379651) and raw reads were deposited in the SRA database under project PRJNA559497 (SRA accessions: SRX6689192–SRX6689197).

To determine the distributions of the viruses in the samples, the viral genomes were clustered using SDT v1.2 [28] with a 98% identity threshold into a viral operational taxonomic unit (vOTU). The reads derived from each swab sample were mapped to a representative of each vOTU using BBMap [29].

### 2.4. Sequence Similarity Network Analysis

The Rep amino acid sequences of the cressdnaviruses identified in this study, together with representative Reps of classified and unclassified cressdnaviruses, were assembled into a cressdnavirus-Rep (cress-Rep) dataset. The cress-Rep dataset was used to infer a sequence similarity network (SSN) using EFI-EST [30] with a similarity score of 60. In the past [8,10,12,31,32], we noted that a similarity score of 60 clusters Reps into cressdnavirus family-level groupings. The resulting Rep amino acid SSN was visualized with an organic layout option in Cytoscape V3.8.2 [33].

### 2.5. Phylogenetic Analyses of the Cressdnaviruses

**Figure 1:**
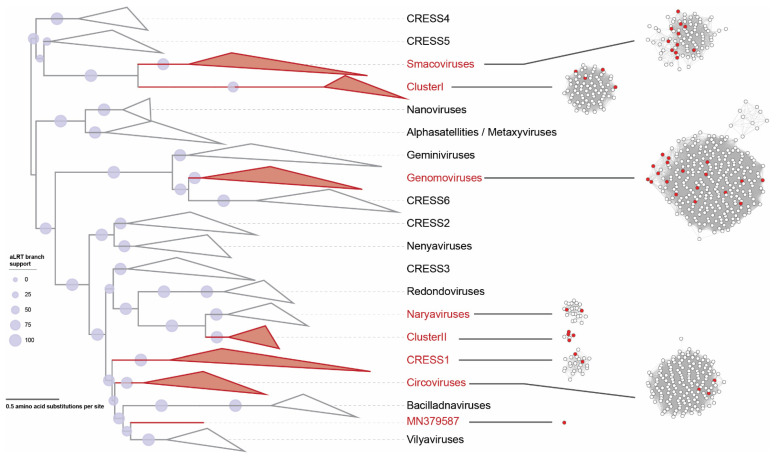
A maximum-likelihood phylogenetic tree of Rep sequences of members of the cressdnavirus families (*Bacillidnaviridae*, *Circoviridae*, *Geminiviridae*, *Genomoviridae*, *Nanoviridae*, *Redondoviridae* and *Smacoviridae*), Alphasatellitidae and Metaxyviridae, CRESS groups 1–6 (unclassified) and putative family-level virus groups naryavirus, nenyavirus and vilyavirus. To the right of the phylogenetic tree, a sequence similarity network of the Rep sequences of the cressdnaviruses determined using EFI-EST [30] is shown. Nodes with red fill represent Rep sequences of viruses from the six chicken samples. Thirteen Reps sequences do not cluster with established virus families in the phylum Cressdnaviricota but form four putative family level clusters (clusterI, clusterII, CRESS1, naryavirus) and one is a singleton.

A representative dataset of Rep amino acid sequences of established families in the phylum *Cressdnaviricota* (*Bacillidnaviridae*, *Circoviridae*, *Geminiviridae*, *Genomoviridae*, *Nanoviridae*, *Redondoviridae* and *Smacoviridae*) [7,34], *Alphasatellitidae* and *Metaxyviridae* [34,35,36] was assembled. To this, we also added Rep amino acid sequences of the groups (CRESS1-6) identified in Kazlauskas et al. [37,38] and those identified by Kinsella et al. [11] (naryavirus, nenyavirus and vilyavirus). Finally, to this Rep dataset, we added the sequences of the two clusters (ClusterI and ClusterII) from the SSN network (Figure 1) and all the Reps from this study. This Rep dataset was aligned with MAFFT v7.113 AUTO mode [39] and the resulting alignment was trimmed with TrimAL [40] using a 0.2 gap threshold. The trimmed alignment was used to infer a maximum-likelihood phylogenetic tree with IQtree 2 [41] with automatic model selection (the best-fit model identified was Q.pfam + F + G4) and aLRT branch support. The phylogenetic tree was visualized with iTOL v5 [42].

The Rep amino acid sequences within clusters of the cressdnavirus in the sequence similarity network were extracted and each cluster level set of sequences was aligned using MAFFT v7.113 AUTO mode [39] and maximum-likelihood phylogenetic trees inferred using PhyML 3.0 [43] with the best fit models, determined using ProtTest 3 [44]. Branches with aLRT support of <0.8 were collapsed using TreeGraph2 [45].

The phylogenetic trees were visualized with iTOL v5 [42].

### 2.6. Phylogenetic Analyses of the Microviruses

The major capsid protein (MCP) amino acid sequences of the microviruses deposited in GenBank and those from this study were aligned using PROMALS3D [46], and this alignment was trimmed with TrimAL [40] with the gappyout option. The trimmed alignment was used to infer a maximum-likelihood phylogenetic tree with FastTree 2 with default settings [47] and visualized with iTOL v5 [42].

## 3. Results and Discussion

### 3.1. Identification of Circular Single Stranded DNA Viruses in Swab Samples

In the *de novo* assembled contigs, 665 contigs were found to be viral-like sequences (751–14,239 nt in length; Table 2). A large number of these are bacteriophage sequences that are most closely related (based on BLASTx analysis) to the viruses in the viral families *Ackermannviridae*, *Autographiviridae*, *Demerecviridae, Herelleviridae*, *Inoviridae*, *Microviridae*, *Myoviridae*, *Podoviridae* and *Siphoviridae* (Table 2). On the other hand, the eukaryotic-infecting viral-like sequences are most closely related to those in the families *Anelloviridae*, *Circoviridae*, *Genomoviridae*, *Parvoviridae* and *Smacoviridae*, and 109 to unclassified cressdnaviruses (Table 2).

Of the 665 viral-like contigs, 134 were determined to be circular based on terminal redundancy. Of these 134 circular viral-like contigs, 38 were determined to be viral-like circular molecules since they only encoded a single viral-like protein (GenBank accessions MN379546–MN379583), and thus, could be sub-genomic molecules. We examined these for common intergenic regions such as in the case of multipartite ssDNA viruses, e.g., nanoviruses [48], as well as some novel cressdnaviruses [8,49] but did not detect any.

The remaining 96 circular contigs can be broadly labelled as cressdnaviruses (*n* = 70) and microviruses (*n* = 26) based on BLASTx similarity. In some of the cases, the circular viral contigs derived from multiple samples were >99% similar and, thus, for the purpose of this study, we used a 98% pairwise identity threshold to determine a unique virus operational taxonomic unit (vOTU). Based on this, there are 67 unique vOTU circular contigs representing cressdnaviruses (*n* = 44) and microviruses (*n* = 23). The raw reads were deposited in SRA databases under project number PRJNA559497 and the *de novo* assembled sequences determined to be circular were deposited in GenBank with accession numbers MN379546–MN379651.

*Cressdnaviricota* is a recently established phylum of ssDNA viruses [7]. A common feature of the members of this phylum are the homologous Rep proteins that have two conserved domains, the HUH superfamily rolling-circle replication endonuclease domain and a superfamily 3 (SF3) helicase domain [50]. Currently, the phylum has two classes, *Repensiviricetes* and *Arfiviricetes*. Within the class, *Repensiviricetes* are the families *Geminiviridae* and *Genomoviridae*, and in *Arfiviricetes* are the families *Bacilladnaviridae*, *Circoviridae*, *Nanoviridae*, *Metaxyviridae*, *Redondoviridae* and *Smacoviridae* [7,34]. In all the cressdnaviruses described from the six-chicken sample, we detected all the conserved HUH and SF3 motifs (Table 3). In the case of the genomoviruses, there is a geminivirus Rep sequence (GRS) [51] that is also relatively conserved and found in the genomovirus Reps (Table 3).

*Microviridae* is a family of ssDNA bacteriophages [13,14]. Microviruses that have been cultured and studied are known to infect enterobacteria. Nonetheless, microviruses are a large part of the virome identified in fecal samples and gut samples of various animals [3,15,16,18,19,20,52]. *Microviridae* has two subfamilies, *Bullavirinae* and *Gokushovirinae*. In general, the MCP of the microviruses is relatively more conserved than the replication initiator protein.

To determine the family level assignment of the cressdnaviruses from the chicken samples, we undertook a sequence similarity network analysis with a network threshold of 60. Three of the viruses could be assigned to the family *Circoviridae*, 17 to *Genomoviridae* and 11 to *Smacoviridae*. Thirteen could not be classified into any established families (Figure 1, Table 3), although some of these do fall into CRESS1 [7,37,38] and naryavirus [11] groups.

**Figure 2:**
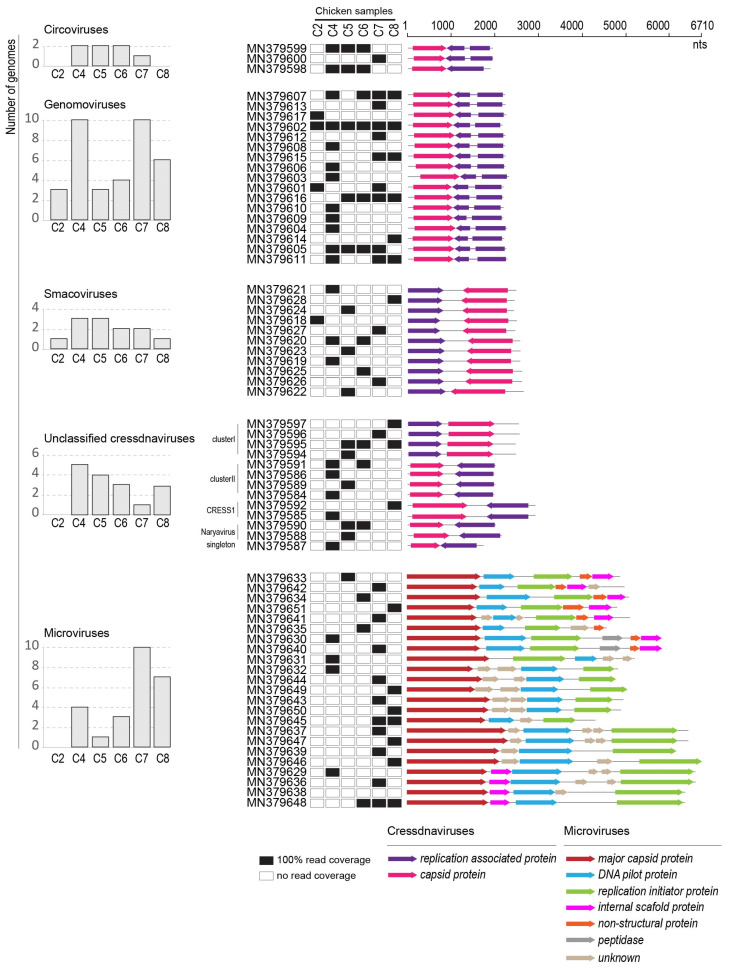
Genome organization of the cressdnaviruses (families *Circoviridae*, *Genomoviridae* and *Smacoviridae* as well as those that are unclassified) and microviruses. Distribution of the virus sequences across the chicken swab samples is shown with black filled boxes. A bar graph summarizes the number of viruses identified in each sample at a family level and those that are unclassified within the phylum *Cressdnaviricota*.

The genome organization and distribution of the viruses identified in the chicken swab samples is illustrated in Figure 2 in a linear form. Out of the six samples, C4 and C7 contained the highest number of detected viral genomes overall, whereas C2 contained the least. Many of the viral genome sequences were present in more than one sample, indicating that these may be commonly circulating in this environment or in the poultry population. One genomovirus (accession number MN379602), for example, was present it all six samples (Figure 2).

A summary of the reads mapped to the vOTUs as well as the depth of coverage for each sample is provided in Supplementary Table S1. We can rule out reagent cross contamination as this was not identified in another sample that was part of the same library preparation and Illumina sequencing run (Supplementary Table S1).

### 3.2. Cressdnaviruses

#### 3.2.1. Circoviruses

The family *Circoviridae* comprises two genera: *Circovirus* whose members have been widely studied due to their impact on the animals in which they cause disease (e.g., beak and feather disease virus and porcine circovirus); and *Cyclovirus* whose members have been found in a variety of samples, but their hosts or biology is unknown [53,54]. Cycloviruses have also been identified in samples of children with and without acute flaccid paralysis [55] and respiratory infections [56], as well as their cerebral fluid [57]. The main distinguishing feature between members of the genera *Circovirus* and *Cyclovirus* is the genome organization of the *rep* and *cp* genes relative to the nonanucleotide motif. The orientation of the genes relative to origin of replication coupled with the Rep amino acid phylogeny is commonly used to distinguish members of the family *Circoviridae* [54]. Members of the family *Circoviridae* are classified into species based on their genome-wide pairwise identity with a species demarcation threshold of 80% [54].

Cycloviruses in chickens were found to be associated with transmissible viral proventriculitis (TVP) that resulted in lesions, runting, and stunting [58]. Lima et al. [59] identified cycloviruses in malabsorption syndrome from chickens in Brazil. Yan et al. [58] detected cycloviruses in chickens with viral proventriculitis in China. Li et al. [60] also identified cycloviruses in the tissues of chickens, and other animals, including goats, cows, and bats, suggesting cross-species transmission of circoviruses and cycloviruses among farm animals.

**Figure 3:**
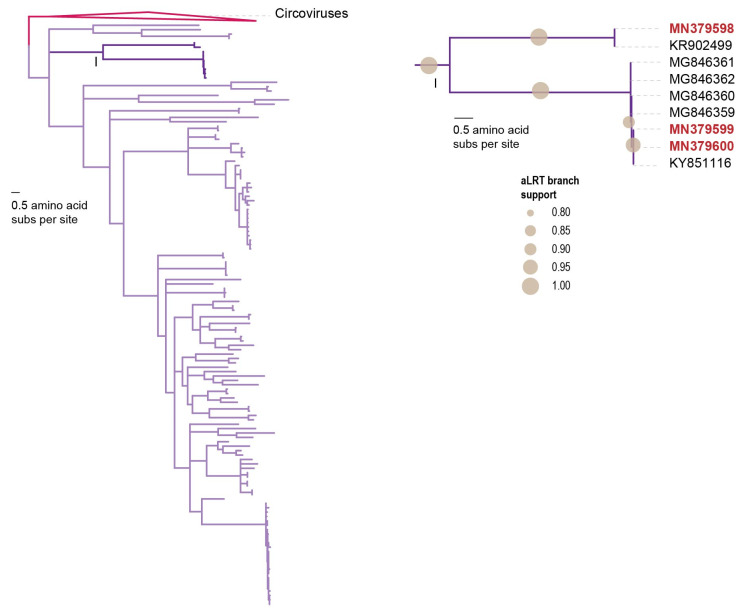
Maximum-likelihood phylogenetic tree of the Rep amino acid sequences of the three unique viruses that belong to the genus *Cyclovirus* (family *Circoviridae*). The maximum-likelihood phylogenetic tree is rooted with representative sequences of Rep sequences in the genus *Circovirus*. Branches with <0.8 aLRT support are collapsed. The clade with the Reps of the cycloviruses from this study is expanded and shown on the right with accession numbers in red bold font.

Here, we report on three cycloviruses identified in three of the chicken samples (Figure 2 and Figure 3). Two of the cycloviruses (accession numbers MN379598 and MN379599) were found in three samples (C4, C5 and C6) whereas one with accession number MN379600 was found in C7. These all have ~1.7 kb genomes and two of the cycloviruses (accession numbers MN379599 and MN379600) have putative spliced reps (Figure 2). The three cycloviruses share 59.3–96.7% genome-wide pairwise identity with each other and 55–96% with other published cyclovirus sequences. The cycloviruses with accession numbers MN379599 and MN379600 share 97% genome-wide pairwise identity with each other and 94.8–96.0% with other cycloviruses with accession numbers KY851116 and MG846359–MG846362 (species *Duck-associated cyclovirus 1*) (Table 3) that are from duck and chicken samples [59,61]. On the other hand, the cyclovirus with accession number MN379598 shares 85.2% pairwise identity with that from a horse sample (accession number KR902499) [62] and belongs to the species *Horse-associated cyclovirus 1* (Table 3). The three cycloviruses identified here form a well-supported clade in the Rep amino acid sequence phylogeny (Figure 3).

#### 3.2.2. Genomoviruses

The family *Genomoviridae* has 10 established genera (*Gemycircularvirus*, *Gemyduguivirus*, *Gemygorvirus*, *Gemykibivirus*, *Gemykolovirus*, *Gemykrogvirus*, *Gemykroznavirus*, *Gemytondvirus*, *Gemytripvirus* and *Gemyvongvirus*) [63]. Two genomoviruses have been found to infect fungi [64,65] and the rest have been identified in a variety of different sample types with no definite hosts. Members of the *Genomoviridae* family are classified based on the Rep amino acid sequence phylogeny for genera assignment and a genome-wide pairwise identity threshold of 78% for species demarcation [53]. Genomoviruses represent a large group of viruses that are widespread and were recently found in feces of many animal species, including birds such as mallard, robins, finches, chicken and black birds [12,63]. Genomoviruses have been detected in chicken samples from New Zealand [66] and Brazil [59,67].

**Figure 4:**
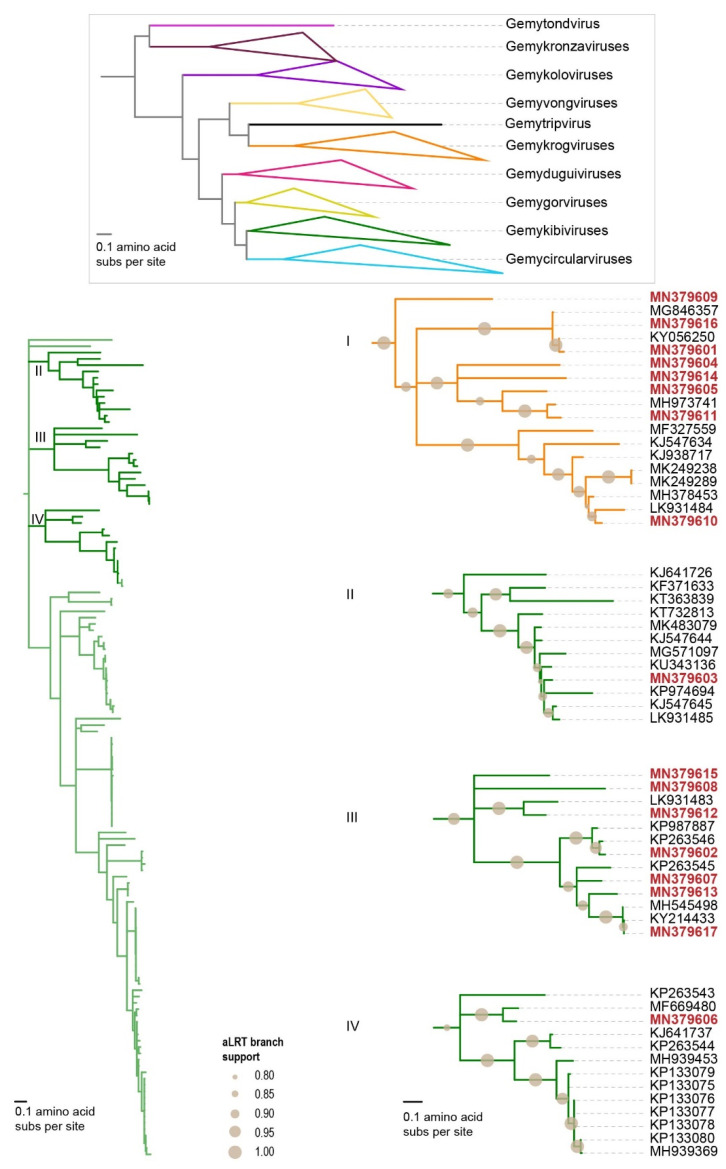
Maximum-likelihood phylogenetic tree of the Rep amino acid sequences of the seventeen unique genomoviruses that belong to the *Gemykibivirus* (*n* = 9) and *Gemykrogvirus* (*n* = 8) genera. The maximum-likelihood phylogenetic tree is rooted with representative Rep sequences of viruses in the family *Geminiviridae*. Branches with <0.8 aLRT support are collapsed. The clades with the Reps of gemykibiviruses and gemykrogviruses from this study are expanded and shown on the right with accession numbers in red bold font.

The genomoviruses (*n* = 17) from this study fall in the genera *Gemykibivirus* (*n* = 9) and *Gemykrogvirus* (*n* = 8) based on the Rep sequence phylogeny (Figure 4). These genomoviruses share >61% genome-wide identity among themselves and with those available in GenBank. Based on their genome-wide pairwise identities, the nine gemykibiviruses were classified into eight species, namely *Gemykibivirus anima1*, *Gemykibivirus cowchi1*, *Gemykibivirus galga1*, *Gemykibivirus galga2*, *Gemykibivirus galga3*, *Gemykibivirus humas1*, *Gemykibivirus humas3* and *Gemykibivirus monas1* [63]. Of the eight, three are new species (*Gemykibivirus galga1*, *Gemykibivirus galga1*, *Gemykibivirus galga3*) established to classify the new gemykibiviruses from the chicken samples. On the other hand, the eight gemykrogviruses were classified into seven species, namely *Gemykrogvirus apime1*, *Gemykrogvirus carib1*, *Gemykrogvirus galga1*, *Gemykrogvirus galga2*, *Gemykrogvirus galga3*, *Gemykrogvirus galga4* and *Gemykrogvirus galga5* [63]. Five of the species (*Gemykrogvirus galga1*, *Gemykrogvirus galga2*, *Gemykrogvirus galga3*, *Gemykrogvirus galga4* and *Gemykrogvirus galga5*) in the genus *Gemykrogvirus* were established to accommodate the genomoviruses from this study (Table 3).

Of note are two genomoviruses (accession numbers MN379616 and MN379617) that are highly similar, sharing 98% and 99% genome-wide pairwise identity to genomoviruses recovered from chicken dung flies (*Fannia* sp.; accession number MH545498) [68] and chicken feces (accession number MG846357) [59], respectively. Furthermore, MN379601 shares ~96% genome-wide pairwise identity with a genomovirus (accession number KY056250) in chicken samples from Brazil [67]. These genomoviruses may represent a group that are associated with fungal species that are commonly found in poultry farms. The distribution of the genomoviruses varies with one (accession number MN379602) being present in all samples, and three (accession numbers MN379605, MN379606 and MN379616) present in at least four of the six samples (Figure 2).

#### 3.2.3. Smacoviruses

The members of the family *Smacoviridae* are classified in twelve genera (*Bovismacovirus*, *Bonzesmacovirus*, *Bostasmacovirus*, *Bovismacovirus*, *Cosmacovirus*, *Dragsmacovirus*, *Drosmacovirus*, *Felismacovirus*, *Huchismacovirus*, *Inpeasmacovirus*, *Porprismacovirus* and *Simismacovirus*) [69]. Smacoviruses have been discovered by metagenomic analyses of diverse animal fecal samples, domestic animal serum and tracheal swab samples and insect samples. No definite host has been determined for smacoviruses but a study by Diez-Villasenor and Rodriguez-Valera [70] suggested gut-associated methanogenic archaea as putative hosts based on CRISPR spacers matching smacovirus-like sequences. As with the classification of genomoviruses, the Rep amino acid sequence phylogeny is used for genus assignment and a 77% genome-wide pairwise identity is used as a species demarcation threshold [71].

**Figure 5:**
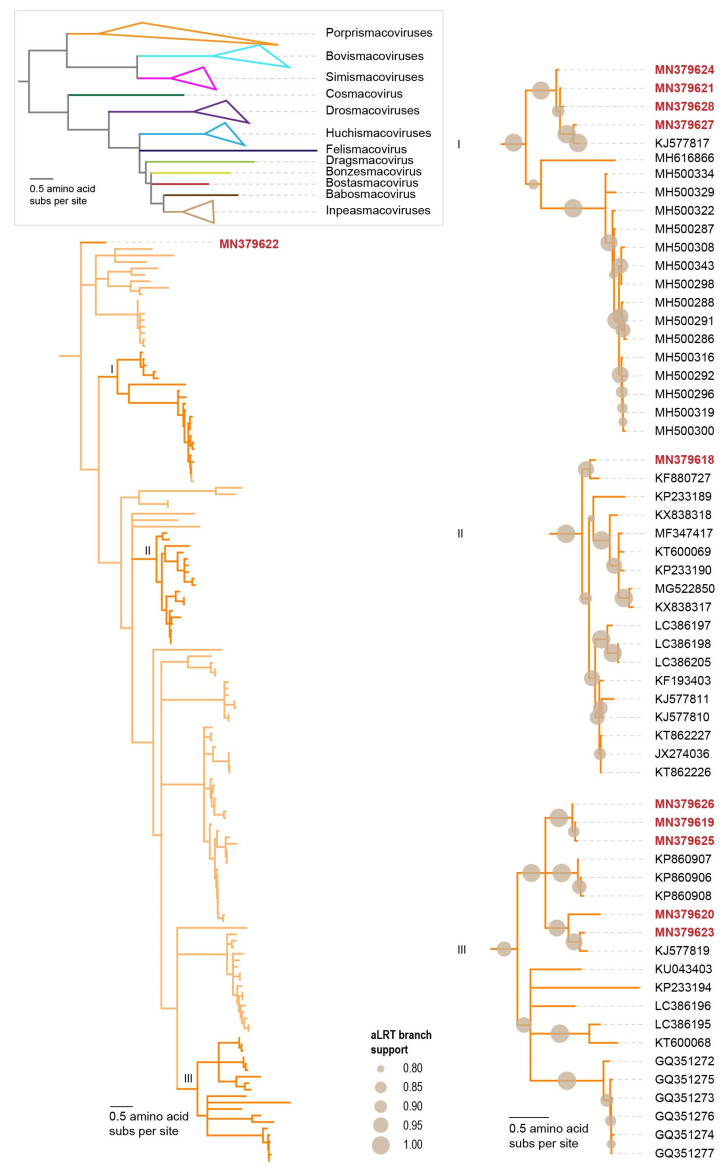
Maximum-likelihood phylogenetic tree of the Rep amino acid sequences of the eleven unique viruses that belong to the *genus Porprismacovirus* (family *Smacoviridae*). The maximum-likelihood phylogenetic tree is rooted with representative Rep amino acid sequences of viruses in the family *Nanoviridae*. Branches with <0.8 aLRT support are collapsed. The clades with the Reps of porprismacoviruses from this study are expanded and shown on the right with accession numbers in red bold font.

Based on the Rep amino acid phylogeny, the 11 smacoviruses from this study are part of the *Porprismacovirus* genus (Figure 5) and share 60–97% genome-wide identity amongst themselves, and 57–75% with all other smacovirus sequences available in GenBank. The 11 smacoviruses are classified into seven species, i.e., *Porprismacovirus chicas2*, *Porprismacovirus chicas3*, *Porprismacovirus chicas4*, *Porprismacovirus chicas5*, *Porprismacovirus chicas6*, *Porprismacovirus chicas7* and *Porprismacovirus turas1* (Table 3). Six of these (*Porprismacovirus chicas2*, *Porprismacovirus chicas3*, *Porprismacovirus chicas4*, *Porprismacovirus chicas5*, *Porprismacovirus chicas6* and *Porprismacovirus chicas7*) are new species established to classify the ones discovered in this study [69]. The only other smacoviruses identified in chicken samples are from Brazil [59,67] and part of the *Huchismacovirus* (*n* = 6) and *Porprismacovirus* (*n* = 2) representing four species (*Huchismacovirus chicas1*, *Huchismacovirus chicas2*, *Huchismacovirus humas1*, *Porprismacovirus chicas1*) [69]. We found only one smacovirus (accession number MN379620) in two samples (C4 and C6), whereas all other are found in individual samples only (Figure 2).

#### 3.2.4. Unclassified Cressdnaviruses

**Figure 6:**
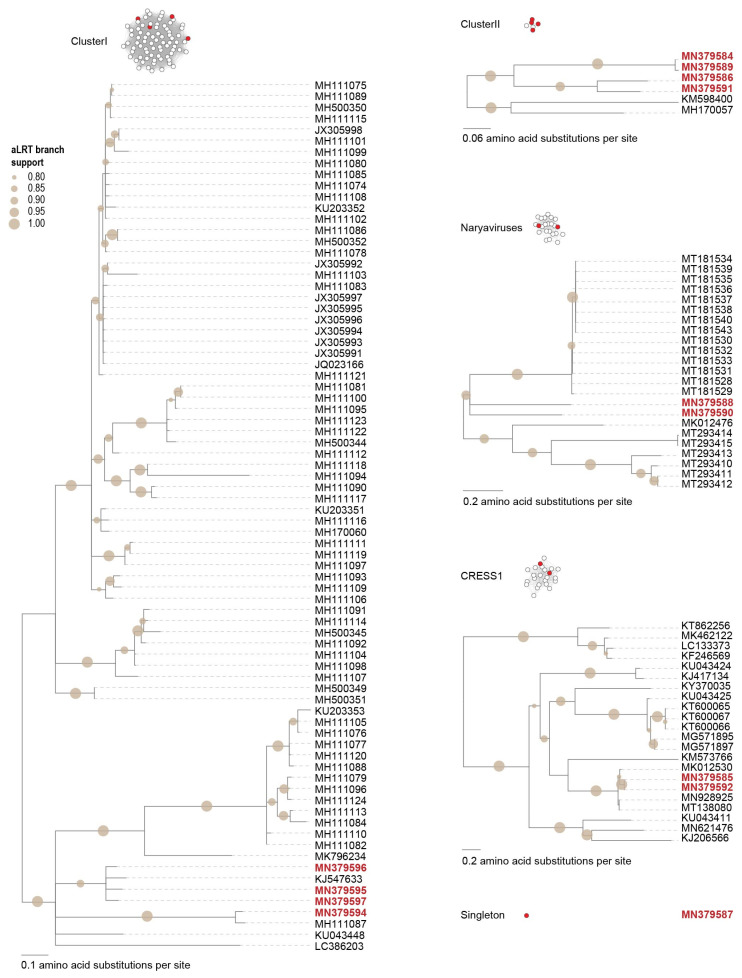
Maximum-likelihood phylogenetic trees (midpoint rooted) for all sequences belonging to the SSN clusters that cannot be assigned to currently established viral families (i.e., ClusterI, ClusterII, CRESS1, naryavirus). Branches with <0.8 aLRT support are collapsed. Accession numbers of Reps encoded by viruses identified in this study are in red bold font.

Many new cressdnaviruses, that are yet to be classified into families, have been discovered over the last decade. Identification of these in poultry samples is no exception [58,59,72]. Here, we have 13 cressdnaviruses that cannot be assigned to any currently established families (Figure 1, Figure 6 and Table 3). These all encode both a Rep and a CP, but they have varied genome organizations (Figure 2). Four genomes have genes that are unidirectionally transcribed and the remaining nine have genes that are bidirectionally organized. They have genomes in the size range of 1690 to 2863 nt (Figure 2).

Based on the SSN analysis, we can group these unclassified cressdnaviruses into four clusters (Figure 1). The four cressdnaviruses in clusterI (accession numbers MN379594–MN379597) are most closely related to those from a sewage-oxidation pond (KJ547633) and a human fecal sample (MH111087) with their Reps sharing >70% amino acid identity (Figure 6).

The second cluster, ClusterII, is relatively small with four Rep sequences from this study (accession numbers MN379584, MN379586, MN379589 and MN379591) and two of the viruses identified in a dragonfly [73] and a porcine serum sample [74] sharing >48% amino acid identity. The Reps of cressdnaviruses with sequence accession numbers MN379584 and MN379589 share 99.3% amino acid identity whereas those of viruses with accession numbers MN379586 and MN379591 share 83.7%. The Reps in this cluster of viruses from the chicken samples cluster together phylogenetically and share >59% identity (Figure 6).

The Reps in third cluster (named naryavirus) share 42–52% amino acid identity with those the two from this study (accession numbers MN379588 and MN379590). The Reps of these two phylogenetically cluster with those of viruses derived from marmot [75] and human fecal samples [11], the latter being associated with Entamoeba [11].

In the last cluster, which included sequences that are part of the CRESS1 group, identified by Kazlauskas et al. [37,38], the Reps of cressdnaviruses with accession numbers MN379585 and MN379592 are 100% identical and group with the Reps of viruses with accession numbers MK012530, MN928925 and MT138080 from turkey, golden pheasant and unknown avian samples [3] sharing >93% identity (Figure 6). The Rep sequences in this cluster share >35% amino acid identity.

The Rep of the singleton with accession number MN379587 (Figure 1) shares ~41% amino acid identity that that of a cressdnavirus with accession number MH973746 discovered in honeybees [16].

It is interesting to note that in sample C2, no unclassified cressdnavirus genome was identified, and only one was identified in sample C7 (Figure 2). The sequence with accession number MN379595 was present in three samples, whereas those with accession numbers MN379590 and MN379591 were present in two samples.

### 3.3. Microviruses

*Bullavirinae* and *Gokushovirinae* are two subfamilies in the family *Microviridae* [13,14]. Within the subfamily *Bullavirinae* there are three genera (*Alphatrevirus*, *Gequatrovirus* and *Sinsheimervirus*) and in *Gokushovirinae* there are four genera (*Bdellomicrovirus*, *Chlamydiamicrovirus*, *Enterogokushovirus* and *Spiromicrovirus*). The well-studied microviruses are known to infect enterobacteria; thus, it is likely that the large number of microviruses that have been identified from various ecosystems and fecal samples of animals likely infect enteric bacteria and these remain largely unclassified.

**Figure 7:**
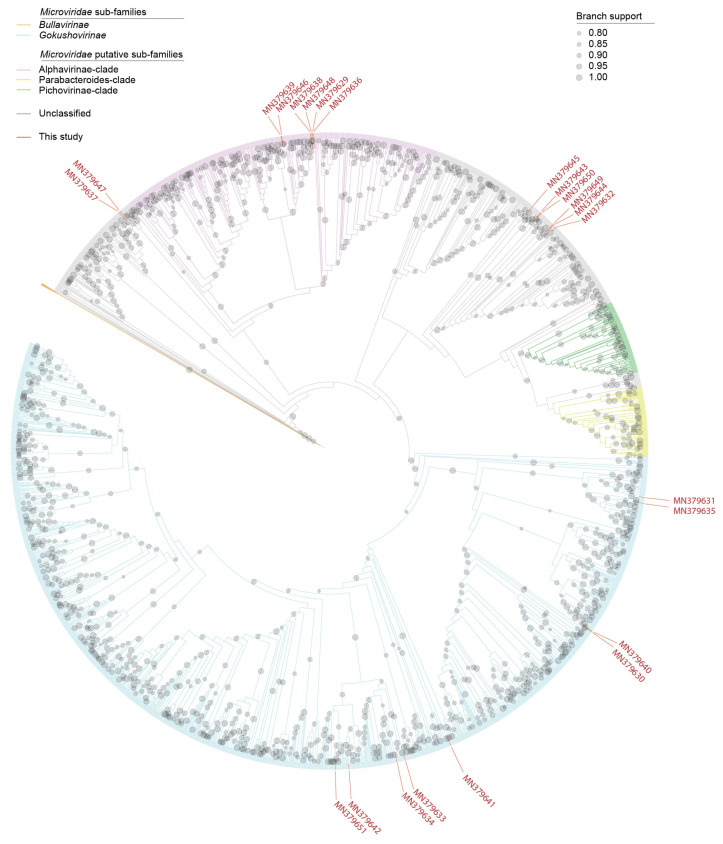
An approximately maximum-likelihood cladogram of the MCP amino acid sequences of microvirus genomes available in GenBank and those identified in this study. The cladogram branches are colored based on sub-families (*Bullavirinae* and *Gokushovirinae*) and Alphavirinae, Parabacteroides and Pichovirinae clades. Branch support with >0.8 aLRT are shown. MCPs of sequences identified in this study are marked with red branches and red colored accession numbers.

Here, we discovered 23 microviruses whose genomes range in size from 4253 to 6710 nt (Figure 2 and Figure 7). In all of these genomes, at least three conserved genes, i.e., those coding for MCP, replication initiator protein and DNA pilot protein (Figure 2) are present. MCP is the most conserved protein amongst all microviruses and in general has been used to roughly assign viruses at a sub-family level. Analysis of phylogeny of the MCP amino acid sequences reveals that nine are likely members of *Gokushovirinae*, six are part of the Alphavirinae-clade and eight part of undescribed clades (Figure 7). The MCP of the microviruses from this study share 45–89% amino acid identity with those of other microviruses available in GenBank. Microviruses with accession numbers MN379639 and MN379646 most closely related to each other sharing ~90% genome-wide identity and their MCPs share 99% amino acid identity.

No microvirus genomes were detected in sample C2 and with the expectation of the ones with accession numbers MN379645 and MN379648, all other microvirus genomes were only identified in a single sample. Sample C7 had ten unique microviruses in it (Figure 2). The microviruses in the chicken samples are likely part of the enteric microbiota of the chicken and, thus, would be infecting the enteric bacterial communities.

## 4. Conclusions

To increase our general knowledge of viruses that infect or are associated with the upper respiratory track of commercial chickens, we undertook shotgun metagenomics sequencing of DNA extracted from buccal swabs. We identified 665 de novo assembled viral-like contigs that share similarities to viruses in the families *Ackermannviridae*, *Anelloviridae*, *Autographiviridae*, *Circoviridae*, *Demerecviridae, Genomoviridae*, *Herelleviridae*, *Inoviridae*, *Microviridae*, *Myoviridae*, *Parvoviridae*, *Podoviridae*, *Siphoviridae* and *Smacoviridae*, and unclassified cressdnaviruses. Of these, 96 were determined to be circular genomes, with 70 being part of the phylum *Cressdnaviricota* (families *Circoviridae*, *Genomoviridae* and *Smacoviridae*) and 26 to be part of the family *Microviridae* in the phylum *Phixviricota*.

The most frequently detected group of viruses across the samples are genomoviruses followed by smacoviruses and unclassified cressdnaviruses. Although only three cycloviruses were identified, two of these were present across three of the same samples. In general, cycloviruses have been found in various sample types but more so in invertebrate samples. Thus, we cannot rule out that these viruses may be associated with invertebrates, such as insects, that are eaten by most avian species. There is limited knowledge about genomoviruses and their hosts, i.e., for at least two that are fungi, it is highly likely that the ones we found in the chicken samples are associated with fungi (either unicellular or multicellular) that inhabit the oropharyngeal region or on the grain and insects that are feed on by the chickens. No host has so far been determined for smacoviruses although researchers, based on CRISPR analysis, have suggested methanogenic archaea as putative hosts. Hence, the smacoviruses from this study may be associated with methanogens that are part of the microbial flora of the tracheal region of the chickens. Four of the unclassified cressdnaviruses appear to be part of the naryavirus group, some of whose members have been found to infect Entamoeba; thus, one can speculate that these could be associated with protists that inhabit the trachea of the chickens. Given the limited knowledge on the various cressdnaviruses, it is not possible to know whether those from this study infect the chickens or are merely infecting organisms that are part of their diet or oral/tracheal microbial flora. The microviruses identified in this study likely infect enterobacteria associated with the oral tract of the chickens.

Many of viruses were found in more than one sample, suggesting that they may be prevalent in chickens. Certainly, further investigation is warranted in to determine the prevalence of these viruses and their pathology, if any, in birds.

## Figures and Tables

**Table 1:** Summary of the families and genera of the classified eukaryote-infecting viruses identified in or associated with chicken.

Genome Type	Family	Genera
dsDNA	*Adenoviridae*	*Atadenovirus, Aviadenovirus, Mastadenovirus, Siadenovirus*
	*Herpesviridae*	*Iltovirus, Mardivirus*
	*Poxviridae*	*Avipoxvirus*
ssDNA	*Anelloviridae*	*Gyrovirus*
	*Circoviridae*	*Cyclovirus*
	*Genomoviridae*	*Gemycircularvirus, Gemykibivirus*
	*Parvoviridae*	*Aveparvovirus, Dependoparvovirus, Protoparvovirus*
	*Smacoviridae*	*Huchismacovirus*
dsRNA	*Birnaviridae*	*Avibirnavirus*
	*Picobirnaviridae*	*Picobirnavirus*
	*Reoviridae*	*Orthoreovirus, Rotavirus*
ssRNA (+)	*Astroviridae*	*Avastrovirus*
	*Caliciviridae*	*Bavovirus, Norovirus*
	*Coronaviridae*	*Deltacoronavirus, Gammacoronavirus*
	*Flaviviridae*	*Flavivirus*
	*Hepeviridae*	*Orthohepevirus*
	*Picornaviridae*	*Aphthovirus, Avisivirus, Enterovirus, Gallivirus, Megrivirus, Orivirus, Sicinivirus, Tremovirus*
ssRNA (-)	*Orthomyxoviridae*	*Alphainfluenzavirus*
	*Paramyxoviridae*	*Avulavirus, Orthoavulavirus, Paraavulavirus*
	*Peribunyaviridae*	*Orthobunyavirus*
	*Pneumoviridae*	*Metapneumovirus*
	*Rhabdoviridae*	*Lyssavirus, Sunrhavirus*
RNA-RT	*Retroviridae*	*Alpharetrovirus, Gammaretrovirus*

**Table 2:** Summary of the number of de novo assembled viral-like contigs (>750 nts in length) from the six samples based on BLASTx analysis. The likely taxonomy assignment of these contigs is based on the top BLASTx hit.

		Contigs in Each Sample						
Family	Length of Contigs (nt)	C2	C4	C5	C6	C7	C8	Total Contigs
*Ackermannviridae*	762–8399	-	-	1	1	-	-	2
*Anelloviridae*	815	-	-	-	-	1	-	1
*Autographiviridae*	1170–1440	1	-	2	-	-	-	3
*Circoviridae*	771–2329	-	2	3	7	2	3	17
*Demerecviridae*	772–2046	-	1	-	-	1	2	4
*Genomoviridae*	760–2347	5	11	4	4	11	8	43
*Herelleviridae*	751–6913	1	-	-	1	3	9	14
*Inoviridae*	867–5794	2	-	-	-	-	2	4
*Microviridae*	781–6787	1	25	16	24	24	36	126
*Myoviridae*	754–8471	23	3	17	11	9	56	119
*Parvoviridae*	785–2429	-	2	-	-	-	-	2
*Podoviridae*	769–14,239	11	1	3	9	9	10	43
*Siphoviridae*	751–11,004	32	6	23	24	10	58	153
*Smacoviridae*	896–2385	3	3	7	4	4	4	25
unclassified cressdnaviruses	768–5193	-	25	20	24	15	25	109

**Table 3:** Summary of the cressdnaviruses identified in this study and the conserved HUH and SF3 motifs in the Rep.

Family	Genus/Group	Species	Accession	Name	Motif I	Motif II	GRS	Motif III	Walker A	Walker B	Motif C
*Circoviridae*	*Cyclovirus*	*Duck-associated cyclovirus 1*	MN379599	Chicken cyclovirus mg5_2967	SWTLNN	PHLQG	-	QNHDYCSK	GASGTGKSRRAA	IIDDF	ITSN
			MN379600	Chicken cyclovirus mg7_102	SWTLNN	PHLQG	-	QNHDYCAK	GASGTGKSRRAA	IIDDF	ITSN
		*Horse-associated cyclovirus 1*	MN379598	Chicken cyclovirus mg4_1122	CFTYNN	KHLQG	-	QNYDYCTK	GETGTGKSRKCA	IIDDF	ITSN
*Genomoviridae*	*Gemykibivirus*	*Gemykibivirus anima1*	MN379613	Chicken genomovirus mg7_74	LFTYSQ	THLHV	RKFDVEDFHPNIVPSL	GGWDYATK	GRSRTGKTILAR	VFDDI	WIMN
			MN379617	Chicken genomovirus mg2_274u	LFTYSQ	THLHV	RKFDVEGFHPNIVPSL	GGWDYATK	GRSRTGKTWLAR	VFDDI	WIMN
		*Gemykibivirus cowchi1*	MN379606	Chicken genomovirus mg4_1196	LLTYAQ	THLHS	DAFDVGGYHPNISPSY	KGFDYTIK	GPSRLGKTLWAR	VFDDI	WLSN
		*Gemykibivirus galga1*	MN379612	Chicken genomovirus mg7_73	LLTYAQ	THLHA	SVFDVAGFHPNISITK	IHYDYAIK	GKSRTGKTNYAR	VFDDI	WISN
		*Gemykibivirus galga2*	MN379615	Chicken genomovirus mg8_401	LLTYSQ	THLHA	DVYDVDGFHPNISPSL	RGYDYAIK	GPTRTGKTMWSR	VFDDV	WLAN
		*Gemykibivirus galga3*	MN379608	Chicken genomovirus mg4_1218	LLTYPQ	NHLHA	DVFDVDGRHPNIQSRL	AGYDYVIK	GDTLTGKTQWAR	IFDDL	YISN
		*Gemykibivirus humas1*	MN379603	Chicken genomovirus mg4_1107	LLTYPQ	IHLHA	RAFDVEGCHPNVSPSR	DGYDYAIK	GPSRMGKTIWAR	IFDDF	WLSN
		*Gemykibivirus humas3*	MN379602	Chicken genomovirus mg2_77	LFTYSQ	THLHA	RKFDVEGFHPNIISTI	GSWDYATK	GPSRTGKTMWAR	VFDDI	WLSN
		*Gemykibivirus monas1*	MN379607	Chicken genomovirus mg4_1210	LFTYSQ	SHLHV	RKFDVEGFHPNIVPSL	GGWDYATK	GPSRTGKTMWAR	VFDDI	WLMN
	*Gemykrogvirus*	*Gemykrogvirus apime1*	MN379611	Chicken genomovirus mg7_70	FLTYSQ	HHFHA	SRLDFGCHHPNIQSVR	RTWDYVGK	GPTRTGKTANIL	VFDDI	MLMN
		*Gemykrogvirus carib1*	MN379610	Chicken genomovirus mg4_1259	IITFPQ	VHYHV	TAFDYFGAHGNIKSVR	KVFDYVGK	GPTRTGKTLYAR	VFDDI	MCMN
		*Gemykrogvirus galga1*	MN379609	Chicken genomovirus mg4_1247	LLTYSQ	THFHV	RLFDFGSSHPNIQIIR	KAFDYAGK	GPTRSGKSVWPR	VFDDL	MCMN
		*Gemykrogvirus galga2*	MN379601	Chicken genomovirus mg2_75	FLTYSQ	SHLHC	SLFDYRGAHPNIKSIR	KPWNYAGK	GPSRTGKTVWAR	IFDDI	MCMN
			MN379616	Chicken genomovirus mg8_416	FLTYSQ	SHLHC	SLFDYRGAHPNIKSIR	KPWNYAGK	GPSRTGKTVWAR	IFDDI	MCMN
		*Gemykrogvirus galga3*	MN379605	Chicken genomovirus mg4_1173	FLTYSQ	CHFHV	SRLDFGGHHPNIQSVR	RVWDYAGK	GPTRTGKTVWAR	VFDDI	MLMN
		*Gemykrogvirus galga4*	MN379614	Chicken genomovirus mg7_78	LLTYSK	VHVHC	FRFDFGGSHPNIRSVS	RTYDYAGK	GPTRTGKTVWAR	VFDDL	CLMN
		*Gemykrogvirus galga5*	MN379604	Chicken genomovirus mg4_1165	LLTYSQ	LHFHC	SRLDYNGSHPNIKPIR	RAWEYTGK	GESRTGKTIWAR	IFDDI	LLCN
*Smacoviridae*	*Porprismacovirus*	*Porprismacovirus chicas2*	MN379619	Chicken smacovirus mg4_881	MITMPR	QHWQC	-	DTWEYETK	PEGNHGKTWLVG	FIDIP	VMTN
			MN379625	Chicken smacovirus mg6_1052	MITMPR	QHWQC	-	DTWDYETK	PEGNHGKTWLVG	FIDIP	VMTN
			MN379626	Chicken smacovirus mg7_57	MITMPR	QHWQC	-	DTWDYETK	PEGNHGKTWLVG	FIDIP	VMTN
		*Porprismacovirus chicas3*	MN379623	Chicken smacovirus mg5_1212	MLTIPR	EHWQV	-	DKWEYETK	PKGNNGKSWLVG	VIDLP	VLTN
		*Porprismacovirus chicas4*	MN379620	Chicken smacovirus mg4_885	MLTIPR	KHWQV	-	EDSDYETK	PKGKAGKSWLIG	IIDMP	VLTN
		*Porprismacovirus chicas5*	MN379627	Chicken smacovirus mg7_67	MLTIPR	DHWQI	-	DKWEYERK	PIGNRGKSWLAG	VIDIP	ILTN
		*Porprismacovirus chicas6*	MN379621	Chicken smacovirus mg4_964	MLTIPR	EHWQV	-	DKWEYERK	PIGNRGKSWLAG	VIDIP	IMTN
			MN379624	Chicken smacovirus mg5_1444	MMTIPR	DHWQV	-	DKWEYEKK	PIGNRGKSWLAG	VIDIP	IMTN
			MN379628	Chicken smacovirus mg8_345	MLTIPR	EHWQI	-	DRWEYERK	PIGNRGKSWLAG	VIDIP	IMTN
		*Porprismacovirus chicas7*	MN379622	Chicken smacovirus mg5_1081	MLTIPQ	KHWQI	-	DVWDYERK	KSGNHGKTWLSQ	IIDIP	IFTN
		*Porprismacovirus turas1*	MN379618	Chicken smacovirus mg2_55	IMTIPQ	EHWQI	-	DECLYERK	AGGNVGKSWFCG	IIDVP	CLTN
unclassified	ClusterI		MN379594	Chicken virus mg5_1345	DATIWI	RHYQF	-	RDFEYVYK	EVGNAGKTAWGM	IIDTP	VLCN
	ClusterI		MN379595	Chicken virus mg6_1197	DATIWC	RHFQF	-	RDFEYVYK	ERGNSGKTAWAM	IIDTP	ILCN
	ClusterI		MN379596	Chicken virus mg7_59	DATIWC	RHFQF	-	RDFEYVYK	ERGNSGKTAWAM	IIDTP	VLCN
	ClusterI		MN379597	Chicken virus mg8_324	DATIWC	RHFQF	-	RDFEYVYK	ERGNSGKTAWAM	IIDTP	VLCN
	ClusterII		MN379584	Chicken virus mg4_280	QLTLNQ	EHIHI	-	QNIDYIRK	GAPGVGKTYSAY	VVEEF	FASV
	ClusterII		MN379591	Chicken virus mg6_2056	QITLNE	KHIHI	-	QNIAYIEK	GDSGSGKTYKAY	VIEEF	IASI
	ClusterII		MN379586	Chicken virus mg4_1578	QITLND	KHIHI	-	QNIDYIEK	GDSGTGKTYKAY	IVEEF	IASI
	ClusterII		MN379589	Chicken virus mg5_2676	QLTLNQ	EHIHI	-	QNIEYIRK	GAPGVGKTYSAY	VVEEF	FASV
	CRESS1		MN379585	Chicken virus mg4_657	CFTSFK	KHLQG	-	KAIEYCKK	GQAGSGKSHHCY	WFDEF	ISTT
	CRESS1		MN379592	Chicken virus mg8_273	CFTSFK	KHLQG	-	KAIEYCKK	GQAGSGKSHHCY	WFDEF	ISTT
	Naryavirus		MN379588	Chicken virus mg5_2197	QITLNE	EHIHC	-	QNVAYVKK	GPSGVGKTERAK	IYDDF	ITSV
	Naryavirus		MN379590	Chicken virus mg5_2876	QLTLNE	EHIHI	-	QNIDYILK	GPSGVGKTNKAL	LYDDF	ITSV
	Singleton		MN379587	Chicken virus mg4_2302	IFTINN	QHLQG	-	QAYNYATK	GDSGTGKSYSAR	VLDEF	ITTN

## Data Availability

Sequences were deposited in GenBank under accession numbers MN379546–MN379651. The raw sequence reads were deposited in SRA under project PRJNA559497 (SRA accessions: SRX6689192–SRX6689197).

## Supplementary Materials

The following are available online at https://www.mdpi.com/article/10.3390/microorganisms9122602/s1, Table S1: Read mapping summary (average fold, genome coverage, based covered and number of reads) to the genomes of the viruses and circular molecules identified in this study.
